# Systematic review of menstrual hygiene management requirements, its barriers and strategies for disabled people

**DOI:** 10.1371/journal.pone.0210974

**Published:** 2019-02-06

**Authors:** Jane Wilbur, Belen Torondel, Shaffa Hameed, Thérèse Mahon, Hannah Kuper

**Affiliations:** 1 International Centre for Evidence in Disability, Department of Clinical Research, London School of Hygiene & Tropical Medicine, London, United Kingdom; 2 Environmental Health Group, Department of Clinical Research, London School of Hygiene & Tropical Medicine, London, United Kingdom; 3 Wateraid, London, United Kingdom; Aga Khan University, PAKISTAN

## Abstract

**Background:**

One quarter of the global population is of menstruating age, yet menstruation is shrouded in discrimination and taboos. Disability also carries stigma, so disabled people may face layers of discrimination when they are menstruating. The objective of the review is to assess the menstrual hygiene requirements of disabled people, the barriers they face, and the available interventions to help them manage their menstruation hygienically and with dignity.

**Methods:**

Eligible studies, gathered across all countries, were identified by conducting searches across four databases (MEDLINE, PubMed, EMBASE, Global Health) in May 2017, with alerts set on each database to highlight new titles added until April 2018. Eligible studies incorporated analyses relevant to menstruating disabled people and/or how their carers provide support during their menstrual cycle.

**Results:**

The 22 studies included were published since 1976; the majority after 2010 (n = 12; 55%). One study was a quasi-experiment; all others were observational. Most studies (n = 15; 68%) were from high income countries and most (n = 17; 77%) focused on people with intellectual impairments, so the review findings focus on this group and their carers. Outcomes investigated include choice and preference of menstrual product, ability to manage menstrual hygiene and coping strategies applied. Barriers faced included a lack of standardised guidance for professional carers; a lack of menstruation training, information and support provided to people with intellectual impairments and their carers; a lack of understanding of severity of symptoms experienced by people with intellectual impairments, the high cost of menstrual products and lack of appropriate options for people with physical impairments. Few interventions were found, and strategies for menstrual hygiene management applied by carers of persons with intellectual impairments include limiting the disabled person’s movements when menstruating and suppressing their menstruation.

**Conclusions:**

Little evidence was identified on the requirements of disabled people and their carers in managing their menstruation, and only one intervention, but a range of barriers were identified. This gap in evidence is important, as the consequences of failing to meet menstrual hygiene needs of disabled people includes shame, social isolation, and even sterilisation.

**Systematic review registration:**

PROSPERO CRD42018095497.

## Introduction

Globally, 663 million people lack access to safe water and 2.4 billion people lack access to adequate sanitation [1]. There is extensive literature showing that disabled people face barriers in accessing appropriate water, sanitation and hygiene (WASH) services in low and middle income countries (LMICs) [2–4]. WASH services are vital for effective menstrual hygiene management (MHM).

UNICEF and the WHO define menstrual hygiene management as *“Women and adolescent girls using a clean menstrual management material to absorb or collect blood that can be changed in privacy as often as necessary for the duration of the menstruation period*, *using soap and water for washing the body as required*, *and having access to facilities to dispose of used menstrual management materials*. *They understand the basic facts linked to the menstrual cycle and how to manage it with dignity and without discomfort or fear*” [5]. Menstrual hygiene management also involves addressing harmful societal beliefs and taboos surrounding the issue [6].

Approximately 75% of people experience premenstrual syndrome (PMS), which includes emotional and physical symptoms that occur between one and two weeks before menstruation [7]. Regular menstruation is a sign of health and fertility; it is inherently female. However, drawing on feminist theory, femininity is linked to beauty, freshness and cleanliness [8]; these are opposed to the qualities associated with menstruation: dirty, bloody and smelly. This means menstruation does not conform to the gender stereotypes, is linked to inferiority and contributes to the devaluation of females [9]. This dichotomy may begin to explain menstrual taboos [9]. These points are demonstrated through the ‘Tampon Experiment’, which aimed to understand how a menstruating woman is perceived by others [8]. When an informed research participant dropped a tampon (a visible reminder than women menstruate) on the floor, she was viewed more negatively by men and women than when she dropped the hair clip (considered a feminine item that is not linked to bodily functions) [8].

Menstrual taboos are rooted in, and drive gender inequality. In some settings menstruating people are viewed as impure, so they are separated from men and banned from using the same water sources in order not to contaminate them [10, 11]. These taboos and social beliefs have led some people to internalise this stigma, reporting that they feel dirty when menstruating and are ashamed of it [12, 13].

Menstruation is not widely spoken about [14, 15]. Many pre-pubescent girls do not receive information about menstruation, so their first menstrual cycle can be a frightening experience. In India, a cross sectional study of 387 school going girls reported that only 37% of them were aware of menstruation before menarche [16].

Affordability of menstrual products is an issue in many countries, especially for people from lower socio-economic groups. In many LMICs, people use bark, paper, sand, mud or cloth to absorb menstrual blood [9]. Evidence exists that some adolescent girls in western Kenya engage in transactional sex to obtain sanitary pads [17–20], contributing to exposure to sexually transmitted diseases [21], pregnancy and school dropout [22].

Attention on MHM has increased over the last decade. Examples include the socio-ecological framework for MHM (developed for school girls and their families) to guide research and interventions in LMICs [23] and the inclusion of MHM in the Sustainable Development Goal 6 [24].

This shift is encouraging, but MHM efforts must be inclusive of disabled people.

This review applies the UN Convention on the Rights of Persons with Disabilities’ (CRPD) definition of disability: *‘Persons with disabilities include those who have long-term physical*, *mental*, *intellectual or sensory impairments which in interaction with various barriers may hinder their full and effective participation in society on an equal basis with others’* [25].

Like menstruation, disability often carries stigma [26]. Research in Uganda and Zambia demonstrate that disabled people are considered ‘dirty’ and contagious, so can be banned from using public latrines and water points [27]. It is likely that disabled people face layers of discrimination when they are menstruating, which will vary for people with different impairment types. Inaccessible latrines means disabled people who cannot stand or see often have to crawl, or sit on dirty latrine seats to change their pads or cloths [10]. People with visual impairments may be unable to identify when their period started and finished [3]. People with hearing, communication or intellectual impairments may be less able to communicate when they are in pain or need support [10]. There is a widespread misconception that disabled people are asexual, so do not receive information on sexual and reproductive health, or menstrual hygiene [10, 26, 28].

### Objectives

The objective of the review is to assess the menstrual hygiene requirements of disabled people, the barriers they face, and the available interventions to help them manage their menstruation hygienically and with dignity. A review protocol is registered online with PROSPERO; registration number: CRD42018095497.

### Disclaimer

This review recognises that gender is a social construct, non-binary and fluid. People who menstruate may identify themselves as male, female, or neither. Therefore, this review uses the terms ‘person’, or ‘people’ who menstruate rather than ‘female’, ‘women’ or ‘girl’, unless these terms are pertinent to the study or theory referenced. The authors also chose to use the terminology ‘disabled people’ rather than ‘people with disabilities’.

## Materials and methods

### Search strategy

The search strategy was designed to identify peer reviewed published studies researching disability and MHM. The review covered all countries; no date limit was set to ensure the widest range of articles could be identified. The searches were conducted in May 2017, with alerts set on each database to highlight new titles added since then. Four online databases were used: MEDLINE, PubMed, EMBASE and Global Health through Ovid SP. Additional relevant studies were identified by reviewing references of included studies and scanning the internet for relevant studies after the database searches were completed. Search terms were generated to encapsulate three main concepts: disability, menstruation and hygiene management. Disability included both specific impairments and broad assessments (e.g. self-reported functional or activity limitations) (S1 Table).

### Inclusion / Exclusion criteria

To be eligible, papers had to in English, published in a peer reviewed journal; be original primary research including experimental, observational and qualitative studies, but excluding economic analyses, systematic reviews, project reports, and policy analysis. No exclusion criteria were set on world region or date of publication. Studies were excluded if they reported no empirical qualitative or quantitative data on MHM and if they analysed disability without the inclusion of MHM and vice versa.

Eligible participants were menstruating disabled persons and/or the carers of disabled persons who provide support during their menstrual cycle. Carers were professionals or family members working in institutions or at home. Disabled persons had specific impairments, activity limitations or self-identified as disabled.

Papers were required to investigate the extent to which disabled people and their carers are able to understand and manage their menstrual cycle hygienically and with dignity. The relevant outcomes explored were purposefully broad as there were anticipated to be limited published studies on the issue. Example outcomes include choice of menstrual management material and preference, ability to manage menstrual hygiene and the menstrual cycle; challenges experienced during menstruation and coping strategies applied; changes in behaviour through the menstrual cycle and its management.

### Study selection

All studies identified through the search process were exported to EndNote version X7. Duplicates were removed. Two authors independently double screened the titles, abstracts and key words against the eligibility criteria. Results were compared and contrasted and full-text records of potentially relevant publications were obtained and screened using the inclusion criteria for final selection of studies for the systematic review.

### Data extraction

Data was extracted from the final selection of studies using pre-designed tables and the socio-ecological framework for menstrual hygiene management [23]. Through the data abstraction process for this review, a number of gaps in the socio-ecological framework in relation to the MHM requirements of disabled people and their carers were identified and additions were made to fill these gaps (Table 1, with changes marked in italic).

**Table 1:** Socio-ecological framework for menstrual hygiene management [23].

Factors that support MHM	Outcomes
**Societal and government policy factors**	Policies, strategies and curriculum; training standards and practices; traditional norms, practices and cultural beliefs
**Environmental and resource availability factors**	Water and sanitation facilities including for solid waste management; availability of affordable, *usable* and culturally appropriate sanitary protection materials
**Interpersonal factors–*disabled person***	Relationship with family, *carer (family and / or professional); relationships with healthcare workers*, teachers and other people in authority; relationships with peers; perceptions of changes in gender roles post-menarche
**Interpersonal factors–*Carer***	Relationship with family, *the disabled person; relationships with healthcare workers* and other people in authority; *relationships with the wider community;* perceptions of changes in gender roles post-menarche
**Personal factors*–disabled person***	Knowledge about the biology of menstruation and MHM, information on menstruation and MHM; skills in coping and behavioural adaptions (including pain relief); attitudes, beliefs and feelings about menstruation* (including sterilisation / long-term contraception); ability to manage menstruation independently*, *and support required*
**Personal factors–*Carer***	Knowledge about the biology of menstruation and MHM, information on menstruation and MHM; skills in coping and behavioural adaptions (including pain relief); attitudes, beliefs and feelings about menstruation* (including sterilisation / long-term contraception); ability to manage another person's menstruation independently*, *support required and caring tasks related to MHM*
**Biological factors**	Menstrual variations due to age and features of menstrual cycle (regular, irregular, heavy, light) and any other biological changes related to menstruation; intensity of menstruation (pain) and influences on behaviour, health and concentration; biological issues that impact on MHM, *such as incontinence*

Data was extracted into Microsoft Excel against the following study and framework components:

1. Publication details: author/s, year, title
2. Study location: low, middle or high-income country, country name
3. Methods: study design
4. Participants: source of participants (household, institution), disability type (e.g. intellectual impairments, physical impairments), means of assessing disability, carer type (family member, professional), sample size
5. Aspect of MHM considered
6. Quality assessment

A meta-analysis was not conducted due to the lack of consistency in study designs, population types and outcomes included. The review was conducted to meet the requirements of the Preferred Reporting Items for Systematic Reviews and Meta-Analyses statement (PRISMA) [29].

### Quality assessment

Studies were assessed for their potential risk of various types of bias, by applying an approach used by Banks et al. [30]. This quality scoring used modified versions of the assessment tools STROBE and RATS for quantitative and qualitative studies [31, 32]. Assessment focused on the risk of potential biases stemming from study design, sampling methods, data collection, data analysis and interpretation. As study methodologies varied widely, papers were evaluated to assess their overall risk of bias instead of applying a rigid cut off criteria. Studies were graded as having a low risk of bias when all or almost of the criteria were fulfilled, and those that were not fulfilled were thought unlikely to alter the conclusions of the study; medium risk of bias when some of the criteria were fulfilled, and those not fulfilled were thought unlikely to alter the conclusions of the study; and high risk of bias when few or no criteria were fulfilled, and the conclusions of the study were thought likely or very likely to alter the conclusions of the study [30] (S2 Table).

## Results

### Study selection

**Fig 1:**
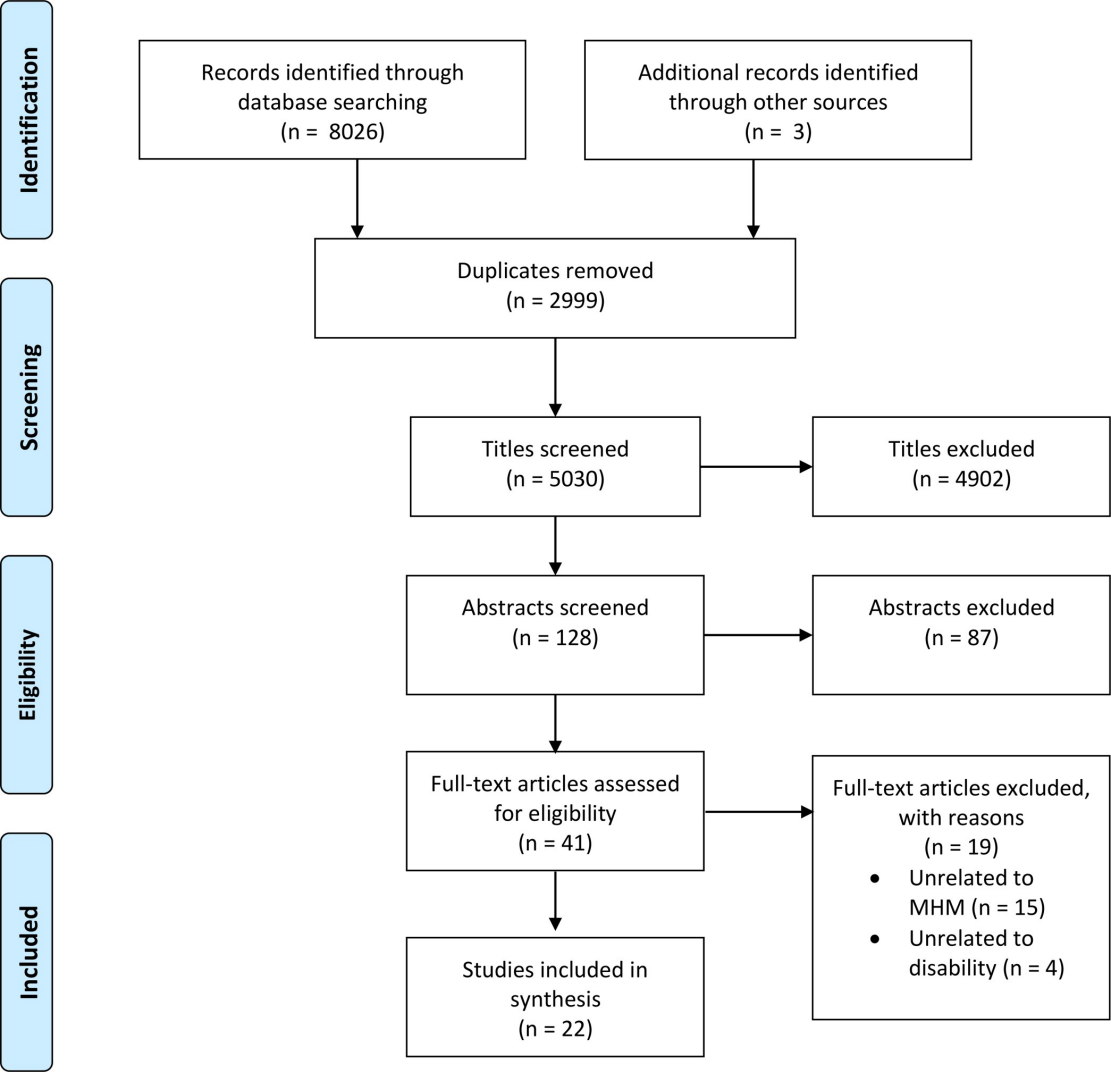
Search strategy with PRISMA flow diagram.

8026 records were identified through database searches. An additional 3 records were sourced through the authors’ knowledge of the available literature. 2999 duplicates were found and removed. An additional 4902 studies were excluded in the title screening process and a further 87 records were excluded through screening the abstracts. 41 full text articles were assessed and 19 were excluded. The remaining 22 studies were included. No additional studies were sourced through database alerts (Fig 1).

### Study characteristics

A summary of the characteristics of included studies are presented in Table 2. Data extracted from all studies against study framework components is captured in Table 3. Studies were published between 1976 and 2017, with the majority published after 2010 (n = 12; 55%). Most of the studies were conducted in high income settings (n = 15, 68%), including Northern Europe (UK, Netherlands and Denmark) (n = 6; 27 Eastern Asia (Taiwan, n = 4, 18%) Northern America (USA and Canada, n = 4, 18%) and Australia and New Zealand (n = 1, 5%). Only seven were conducted in LMICs (32%), including in Southern Asia (India, n = 3, 14%); Eastern Europe (Turkey, Bosnia and Herzegovina, n = 2, 9%); Eastern Africa (Malawi, n = 1, 5%); and South Africa (n = 1, 5%). The majority of studies were quantitative (n = 14; 64%); one study was a quasi-experiment; all others were qualitative.

**Table 2:** Characteristics of included studies.

Variable	Detail	Number	%
*World Bank region*	Low-middle income country	7	32
	High-income country	15	68
*Location*	Northern America	4	18
	Northern Europe	6	27
	Eastern Europe	2	9
	Eastern Africa	1	5
	Southern Africa	1	5
	Eastern Asia	4	18
	Southern Asia	3	14
	Australia and New Zealand	1	5
*Decade of publication*	1970	1	5
	1980	2	9
	1990	1	5
	2000	6	27
	2010	12	55
*Study design*	Qualitative	7	32
	Quantitative—Cross-sectional survey	11	50
	Quantitative—Case-control	3	14
	Quasi-experimental	1	5

**Table 3:** Data extracted against study framework components.

First author	Year	World Bank region	Country	Study Design	Disability sub-group	Disabled person (n)	Carer (n)	Main focus
**Carnaby, S.**	2002	HIC	UK	Qualitative	Intellectual	0	Number not specified in paper	Carers' KAP*
**Charlifue, S.W.**	1992	HIC	USA	Quantitative	Physical	231	0	Menstrual product
**Chou, Y. C.**	2009	HIC	Taiwan	Quantitative	Intellectual	92	0	PMS
**Chou, Y. C.**	2012	HIC	Taiwan	Qualitative	Intellectual	13	12	Carers' KAP
**Patage, D.P.**	2015	LMIC	India	Quantitative	Multiple	198	0	Menstrual product
**Goldstein, H.**	1988	HIC	Denmark	Quantitative	Intellectual	15	0	Menstrual cycle
**Hamilton, A.**	2011	HIC	USA	Quantitative	Intellectual	124	Number not specified in paper	PMS
**Ibralic, I.**	2010	LMIC	Bosnia and Herzegovina	Quantitative	Intellectual	31	0	PMS
**Kirkham, Y. A.**	2013	HIC	Canada	Quantitative	Multiple	300	Number not specified in paper	Menstrual suppression
**Kyrkou, M.**	2005	HIC	Australia and New Zealand	Quantitative	Intellectual	24	0	PMS
**Lin, L. P.**	2011	HIC	Taiwan	Quantitative	Intellectual	0	1152	Menstrual suppression
**Lin, L. P.**	2011	HIC	Taiwan	Quantitative	Intellectual	0	1152	Carers' KAP
**Mason, L.**	2007	HIC	UK	Qualitative	Intellectual	6	53	Training for disabled persons
**Obaydi, H.**	2008	HIC	UK	Qualitative	Intellectual	26	Number not specified in paper	PMS
**Perrin, J. C.**	1976	HIC	USA	Qualitative	Intellectual	20	Number not specified in paper	Menstrual suppression
**Ranganath, P.**	2012	LMIC	India	Quantitative	Intellectual	0	10	PMS
**Rodgers, J.**	2005	HIC	UK	Quantitative	Intellectual	452	217	Training for disabled persons
**Altundağ**	2015	LMIC	Turkey	Quasi-experimental	Intellectual	54	0	Training for disabled persons
**Thapa, P.**	2017	LMIC	India	Qualitative	Intellectual	0	23	Menstrual suppression
**Van der Merwe**	1987	LMIC	South Africa	Quantitative	Multiple	152	0	Menstrual suppression
**van Schrojenstein Lantman-deValk**	2011	HIC	Netherlands	Quantitative	Intellectual	234	0	Menstrual suppression
**White, S.**	2016	LMIC	Malawi	Qualitative	Multiple	36	15	Barriers and outcomes

*Knowledge, attitudes and practices

### Description of studies

Of the 22 studies, disabled persons were the primary research participant in the majority (n = 13; 59%), followed by the carer (n = 6; 27%), or the carer and the disabled person (n = 3; 14%) (Table 4). These participants were sourced through institutions (n = 13; 59%), such as hospitals and residential homes; households (n = 6; 27%) and households and institutions (n = 3; 14%). The means of assessing disability ranged from clinical (n = 8; 36%), self-reported (n = 4; 18%) to government lists (n = 2; 9%). Seventeen (77%) studies focused on people with intellectual impairments, followed by multiple impairments (n = 3; 14%) and physical impairments (n = 2; 9%).

**Table 4:** Characteristics of participants and quality assessment.

Variable	Detail	Number	%
Primary research participants	Carer	6	27
	Disabled person and carer	3	14
	Disabled person	13	59
Source of participants	Household	6	27
	Institution	13	59
	Household and institution	3	14
Means of assessing disability	Clinical	8	36
	Self-reported	4	18
	Government list	2	9
	Not given	8	36
Disability type	Multiple	3	14
	Intellectual	17	77
	Physical	2	9
Quality assessment: risk of bias	Low	13	59
	Medium	7	32
	High	2	9

The quality assessment identified 13 (59%) studies as having low, seven (32%) as medium and two (9%) as high risk of bias. The main reasons for potential bias was the limitations in generalisability of results due to a small sample and response rate being lower than 70%.

### Impacts of menstruation

#### Pre-menstrual symptoms and communication difficulties experienced by people with intellectual impairments

Nine papers (41%) covered pre-menstrual symptoms (PMS) [33–41]. Eight reported PMS symptoms and related behaviour, including menstrual cramps, mood swings, fatigue, irritability, anger, social withdrawal, decreased concentration, increased hyperactivity, self-injury and inappropriate handling of menstrual blood or hygiene products experienced by people with intellectual impairments [34–41]. Six papers assessed the frequency and severity of pain [36–41], three of which compared these between disabled and non-disabled people [37, 38, 41].

Obaydi and Puri stated that PMS was experienced by 92% of the group of people with autism, compared to 11% in the control group of non-disabled people [38]. This study had the lowest risk of bias. Kyrkou also concluded that people with Down syndrome or autism experienced higher rates of pain than the general population [37]. Due to the challenges in communicating the extent and location of pain, Kyrkou deduced this through changes in behavior [37]. However, Ibralic et al. [41] and Ranganath and Ranganath [39] contradicted this finding. Ibralic et al. reported that PMS symptoms were almost equally distributed between non-disabled people and people with an intellectual impairment [41]. Ranganath and Ranganath reported that no one with Down syndrome experienced menstrual pain or premenstrual tension, but the authors did not include an assessment of the participant’s communication abilities or factor this into the results [39]. Ranganath and Ranganath’s study was marked as having a high risk of bias [39].

Three studies investigated the severity of PMS symptoms by disability type [34, 37, 40]. All studies concluded that there is divergence within groups. Kyrkou [37] and Hamilton et al. [40] recognised that the ability to report and locate pain was a determining factor. For instance, within the intellectual impairment group, Kyrkou found that 67% (n = 8) of the research participants with Down syndrome were able to say that they were in pain or point to where they had pain, even those with limited communication abilities [37]. Only one of the nine participants on the autistic spectrum was able to point to, or state when she was in pain, even though all participants had good communication skills.

Three studies stated that the inability of some people with an intellectual impairment to understand the source of pain and communicate affected their behaviour [34, 35, 37].

*"She gets short tempered*. *But it’s not her fault*. *She can’t speak very well*, *so I think that’s how she expresses herself”* (carer from India) [35].

#### Concerns of carers of people with intellectual impairments

Six (27%) studies investigated the key concerns of carers who support people with intellectual impairments [34–37, 42, 43].

Carers (mothers) of people with an intellectual impairment in Thapa and Sivakami’s study in India reported that difficulties with communicating to daughters, and vice versa, were a major challenge [35]. Challenges with communication lead mothers to rely on observing changes in their daughter’s behaviour to anticipate menstruation [34]. Predictors include irritability, restlessness, crying, self-harm, decreased appetite and disruptions in sleeping patterns [34].

Other challenges reported by carers included an aversion to wearing a menstrual product, a lack of adherence to social and cultural norms, such as inappropriate handling of menstrual blood and product, talking to others about their menstruation and changing the used menstrual product in front of others [35–37, 43].

*"She will leave the door open while changing her pad*, *and doesn’t understand that her elder brother is at home*. *So I tell her*, *‘Always bolt this door from inside*.*’ Sometimes she understands*, *but sometimes she starts changing in front of them"* (carer from India) [35]*"*.

One of these six studies investigated professional carers’ levels of satisfaction of intimate care tasks, finding that menstrual care was the second most disliked aspect for residential staff (after giving enemas), and the most disliked aspect for day unit staff (who do not give enemas) [42].

### Strategies for menstrual hygiene management

#### Menstrual product acceptability for people with physical impairments

Four studies (18%) investigated the menstrual product used and preference [34, 35, 44, 45]. Two of these studies considered the product used [44, 45]. One [44] explored the product acceptability from the perspective of people with spinal cord injuries, and the remaining two [34, 35] investigated the carers’ product preference. 19% of the sample in the study focusing on people with spinal cord injuries (conducted in the USA), reported discomfort and difficulty in positioning the menstrual product to ensure its maximum absorbency, as well as increasing difficulties with catheters and urinary management during menstruation [44].

#### Menstrual product acceptability for people with intellectual impairments

Three of the 22 studies explored the disabled person’s preference through the carer [34, 35, 43]. The studies reported that the people with an intellectual impairment often refused to wear the menstrual product, leading to stress felt by the carer and constant negotiation with the disabled person.

*"My biggest problem was that she didn’t want to wear a pad*. *The understanding isn’t there* (carer from England) [43].*”*

In a study, undertaken in India, mothers limited their daughter’s physical movements during menstruation so that she would not go outside with blood stained clothes [35]. Another coping mechanism applied by carers in Taiwan, was sewing the pad into the underwear or buying adult sized nappies for their daughters [34].

In two of the four studies, mothers were caring for daughters with incontinence [34, 35]. These carers felt that menstruation added another layer of complication [35], and that the cost of nappies and pads were a major concern [34]

#### MHM training and support for people with intellectual impairments

Five studies (23%) investigated MHM training given to people with intellectual impairments [35, 37, 43, 46, 47]. One study highlighted a lack of training and support provided to this group because carers did not believe that the individual would understand MHM information [35]. The authors hypothesised that some people with intellectual impairments refused to wear a menstrual product because they were not given any MHM information, including being shown a menstrual product, or practice wearing it prior to their first menstrual cycle. Consequently, they did not understand the purpose of a menstrual product, did not feel comfortable wearing it and associated it with menstrual cramps [35].

One of these fives studies explored the teaching on MHM provided to people with intellectual impairments in institutions [47]. It highlighted a lack of correlation between training provided and the person’s level of understanding [47]. Three studies showed positive correlations between providing MHM training to people with intellectual impairments and an increased ability to manage menstruation independently [35, 37, 46]. In Kyrkou’s study, conducted in Australia and New Zealand, people with Down syndrome who had been given MHM information prior to puberty were better able to cope with their menstrual cycle than those who were not [37]. Altundağ and Calbayram showed in their study in Turkey, that using a doll to practice changing and disposing of a used menstrual product, was an effective way to increase the MHM skills of people with intellectual impairments [46].

#### MHM training and support for carers

Five studies investigated the level of training and support provided to carers (professionals and mothers) on how to manage menstruation of a person with an intellectual impairment [34, 35, 42, 48, 49]. Three of these studies [42, 48, 49] focused on professional carers working in institutions and two studies on mothers at home [34, 35]. The three studies conducted in institutions highlighted limited MHM training and standards for intimate and personal care tasks; that the task’s importance was under-recognised by management; understanding of the menstruation of people with intellectual impairments was low, and support provided on menstrual issues was lower than sex education, but higher than menopause [42, 48, 49].

The mothers in the two studies focusing on care provided within the family were given no guidance, information or support on how to manage their daughter’s menstruation, leaving them feeling overwhelmed and unsupported [34]. In the Indian and Taiwanese settings, mothers believe that menstruation is a private issue so did not discuss their daughter’s menstrual cycle with anyone else, including professionals [34, 35].

#### Menstrual suppression

Six papers included an analysis of menstrual suppression of people with intellectual impairments [34–36, 50–52]. Menstrual suppression includes long-term contraception (i.e. oral contraceptive pill and the patch) and sterilisation (i.e. hysterectomy, tubal ligation). Two of these six studies were from the LMIC and the remaining four studies were from HICs [35, 50]. Two studies [50, 52] were published before 2000 and four after 2010 [34–36, 51].

Of these six papers, five reported that people with intellectual impairments were sterilised or on long-term contraception. Reasons for sterilisation cited by carers including a perception that menstruation care is a “burden”, a fear of unwanted pregnancies [35, 36, 50–52], difficulties related to the menstrual care tasks; the perceived lack of benefit for the person with an intellectual impairment, as well as mothers’ desire not to “burden” an older daughter with the menstrual care tasks when she is no longer able to undertake these tasks [35, 50, 52].

*"I used to do everything–changing the pads every three–four hours*, *taking her to the toilet*. *But she was not aware at all; there were no feelings in her*. *Then when she was 16 years old*, *I realised that I could not do it anymore*, *and it was not benefitting her in anyway*. *Then we got her operated upon*. *We got her surgery done*, *and got her uterus removed”* (carer from India) [35].

Three studies included satisfaction levels of carers post sterilisation [35, 50, 52]. All of these reported high levels of carer satisfaction. One study from Taiwan challenged this trend of menstrual suppression [34]. In this study, regular menstruation was seen as an indication of good bodily health and daughters with an intellectual impairment were given medicine to help regulate their cycles. However, almost all mothers in this study were advised by relatives and medical professionals to sterilise their daughters in order to eliminate the ‘tedious’ menstrual care, for better hygiene and to prevent unwanted pregnancies [34].

## Discussion

Our search sought to identify studies exploring the MHM requirements of disabled persons, but only found 22 studies that met the inclusion criteria. The majority of studies focused on people with intellectual impairments and their carers.

**Fig 2:**
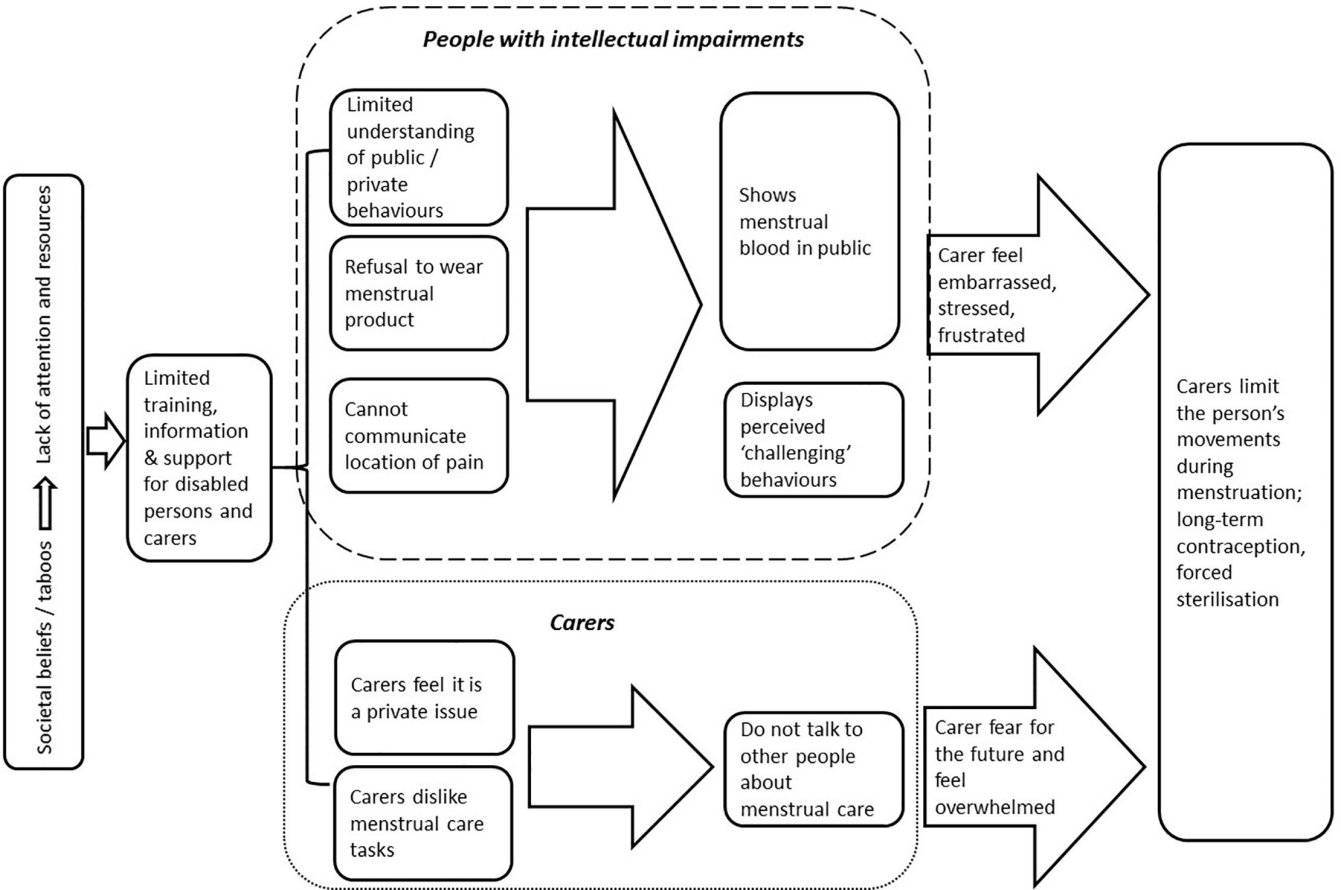
Flow diagram of review findings.

Fig 2 summarises the key findings in this review that relate to people with intellectual impairments and their carers. It shows that societal beliefs and taboos around menstruation and disability means the issue is shrouded in silence, and that it lacks attention and resources. The silence surrounding disabled people’s menstrual hygiene requirements is demonstrated by the limited number of peer reviewed studies gathered for this review. Without rigorous evidence from different contexts, it is difficult to advocate for greater attention and resourcing to meet the MHM requirements of disabled people and their carers. The subsequent dearth of MHM training, information and support tailored to meet disabled people and their carers requirements means some people struggle to manage. Strategies for MHM applied by carers include limiting the disabled person’s movements when menstruating and suppressing their menstruation.

The top box in Fig 2 focuses on people with intellectual impairments. This review has shown that some people with intellectual impairments do not always understand or follow social and cultural norms [35–37, 43], or wear a menstrual product [34, 35]. This group face challenges in understanding PMS and communicating when in discomfort [33–41]. Carers reported subsequent ‘menstrual behaviours’ make them feel stressed, embarrassed and they coped by not letting their daughters leave the home or sought ways to supress their menstruation [35]. The authors propose that if repetitive, accessible MHM information and training is provided regularly to the persons with intellectual impairments, they may get a deeper understanding of cultural and social norms and be better able to manage their menstruation more independently.

The bottom box in Fig 2 focuses on findings related to carers, which highlights an absence of standards and training on providing menstrual care in the institutions covered in the studies [42, 48, 49]. Findings show that professional carers dislike providing menstrual care [42]. If combined, these two factors might mean that a disabled person’s dignity and personal hygiene is compromised in these institutional settings.

The review found that MHM training and support is not provided to family members who care for daughters with intellectual impairments [34], and that mothers also dislike providing menstrual care [35, 50, 52]. Mothers reported an inability to see how menstruation benefit their daughters [35], which is intertwined with the societal belief that disabled people should not be parents or sexual beings [53]. Disability and menstruation related taboos discourages open dialogue, meaning mothers do not seek advice or support, because they view the provision of menstrual care as a private issue [34, 35].

In addition to the findings captured in Fig 2, included studies also investigated the disabled person’s preference of menstrual product [34, 35, 44, 45]. Research participants, with a physical disability, reported low levels of satisfaction with the menstrual product used (sanitary pads with and without tampons), stating that they find the products uncomfortable, difficult to place and use with catheters [44].

### Implications for future research

There is limited evidence about the MHM requirements of disabled people, interventions to meet these and an assessment of their impact. This is particularly stark in LMICs, so research to investigate these topics must be carried out in these settings. Another key research gap is around the development of standardised measurements of MHM related outcomes for disabled people and their carers [54], and here the socioecological framework for MHM, adapted to include disabled persons and their carers (Table 1), could be a start. It is useful as the framework recognises MHM outcomes have individual, social and environmental influences that affect menstrual experiences and MHM among the target population.

More research is required to explore the severity of PMS experienced by disabled people compared to non-disabled people, and compared within disability groups with the view of developing mechanisms that enable disabled people to better locate and communicate pain. Finally, research on menstrual product preference and effectiveness for people with different impairments, to understand if the current products on the market are suitable and acceptable, should also be conducted.

### Review strengths and limitations

To the authors’ knowledge, this is the first systematic review of the MHM requirements of disabled people and their carers. This review restricted the search to studies in the English language and the number of datasets (such as excluding the CINAHL database), so some relevant studies may have been missed. Few studies met the inclusion criteria and across those, there was no standardised measurement of outcomes, meaning an outcome assessment across the studies was difficult. For instance, only seven (32%) studies defined symptoms and practices associated with menstruation, and only 12 (55%) studies identified the means for assessing disability.

There were not enough studies with consistent methods for a meta-analysis. The authors mitigated this by using Banks et al.’s [30] quality assessment that combines the STROBE and RATS assessment tools for quantitative and qualitative studies [31, 32]. The main reasons for risk of bias are due to a sample size being smaller than 100 and the response rate being less than 70%, or not reported. This could lead to an over estimation of impacts. However, there are no great divergences between the findings in papers that have a high risk of bias and those with a medium or high risk of bias, which alleviates concerns.

### Conclusion

In conclusion, limited evidence was identified on the MHM requirements of disabled people and their carers, though a number of barriers were identified. This evidence gap is important and must be filled with future research. MHM interventions that address these barriers must be developed, tested and scaled up in partnership with disabled people. If the inaction continues, disabled people’s rights will continue to be violated; they will continue to face social exclusion and potentially sterilisation.

## Supporting information

S1 Checklist PRISMA checklist. (PDF)

S1 Table Search string for PubMed. (PDF)

S2 Table Quality assessment for all studies. (PDF)

S3 Table Summarised data extraction table. (PDF)
