# Supplementary material for: Systematic review of menstrual hygiene management requirements, its barriers and strategies for disabled people
Source: PLoS One. 2019 Feb 6;14(2):e0210974. doi: 10.1371/journal.pone.0210974 (PMC6365059; doi:10.1371/journal.pone.0210974)
Supplement: S1 Table — (PDF) [file pone.0210974.s002.pdf]

S1 Table. Search String for PubMed

1	developing countr*[MH] OR developing countr*[TIAB] OR developing nation*[TIAB] or developing world[TIAB]
2	least developed countr*[TIAB] OR least developed nation*[TIAB] OR least developed world[TIAB] OR least-developed countr*[TIAB] OR least-developed nation*[TIAB] OR less-developed countr* OR less-developed nation*[TIAB] OR less developed countr*[TIAB] OR less developed nation*[TIAB]
3	under-developed countr*[TIAB] OR under developed countr*[TIAB] OR underdeveloped countr*[TIAB] OR under-developed nation*[TIAB] OR under developed nation*[TIAB] OR underdeveloped nation*[TIAB] OR under-developed world[TIAB] OR under developed world[TIAB] OR underdeveloped world[TIAB] OR under-developed econom*[TIAB] OR under developed econom*[TIAB] OR underdeveloped econom*[TIAB]
4	third world countr*[TIAB] OR third world nation*[TIAB] OR third-world countr*[TIAB] OR third-world nation*[TIAB]
5	low- and middle-income countr*[TIAB] OR low and middle income countr*[TIAB] OR low- and middle-income nation*[TIAB] OR low and middle income nation*[TIAB] OR low- and middle-income world[TIAB] OR low and middle income world[TIAB] OR low- and middle-income econom*[TIAB] OR low and middle income econom*[TIAB] OR low income countr*[TIAB] OR middle income countr*[TIAB] OR low-income countr*[TIAB] OR middle-income countr*[TIAB]OR low income nation*[TIAB] OR middle income nation*[TIAB] OR low-income nation*[TIAB] OR middle-income nation*[TIAB] OR low income world[TIAB] OR middle income world[TIAB] OR low-income world[TIAB] OR middle-income world[TIAB] OR low income econom*[TIAB] OR middle income econom*[TIAB] OR low-income econom*[TIAB] OR middle-income econom*[TIAB]
6	LIC[TIAB] OR LICs[TIAB] OR MIC[TIAB] OR MICs[TIAB] OR LMIC[TIAB] OR LMICs[TIAB] OR LAMIC[TIAB] OR LAMICs[TIAB] OR LAMI countr*[TIAB]
7	Transitional countr*[TIAB] OR Transitional econom*[TIAB] OR Transition countr*[TIAB] OR Transition econom*[TIAB]
8	Asia[MH] OR Africa[MH] OR South America[MH] OR Caribbean region[MH] OR Central America[MH]
9	Afghanistan[TIAB] OR Albania[TIAB] OR Algeria[TIAB] OR American Samoa[TIAB] OR Angola[TIAB] OR Antigua[TIAB] OR Barbuda[TIAB] OR Argentina[TIAB] OR Armenia[TIAB] OR Azerbaijan[TIAB] OR Bangladesh[TIAB] OR Belarus[TIAB] OR Byelarus[TIAB] OR Byelorussia[TIAB] OR Belorussia[TIAB] OR Belize[TIAB] OR Benin[TIAB] OR Bhutan[TIAB] OR Bolivia[TIAB] OR Bosnia[TIAB] OR Herzegovina[TIAB] OR Hercegovina[TIAB] OR Bosnia-Herzegovina[TIAB] OR Bosnia-Hercegovina[TIAB] OR Botswana[TIAB] OR Brazil[TIAB] OR Brasil[TIAB] OR Bulgaria[TIAB] OR Burkina[TIAB] OR Upper Volta[TIAB] OR Burundi[TIAB] OR Urundi[TIAB] OR Cambodia[TIAB] OR Republic of Kampuchea[TIAB] OR Cameroon[TIAB] OR Cameroons[TIAB] OR Cape Verde[TIAB] OR Central African Republic[TIAB] OR Chad[TIAB] OR Chile[TIAB] OR China[TIAB] OR Colombia[TIAB] OR Comoros[TIAB] OR Comoro Islands[TIAB] OR Comores[TIAB] OR Congo[TIAB] OR DRC[TIAB] OR Zaire[TIAB] OR Costa Rica[TIAB] OR Cote d'Ivoire[TIAB] OR Ivory Coast[TIAB] OR Cuba[TIAB] OR Djibouti[TIAB] OR Obock[TIAB] OR French Somaliland[TIAB] OR Dominica[TIAB] OR Dominican Republic[TIAB] OR Ecuador[TIAB] OR Egypt[TIAB] OR United Arab Republic[TIAB] OR El Salvador[TIAB] OR Eritrea[TIAB] OR Ethiopia[TIAB] OR Fiji[TIAB] OR Gabon[TIAB] OR Gabonese Republic[TIAB] OR Gambia[TIAB] OR Georgia[TIAB] OR Ghana[TIAB] OR Gold Coast[TIAB] OR Grenada[TIAB] OR Guatemala[TIAB] OR

	Guinea[TIAB] OR Guinea-Bissau[TIAB] OR Guiana[TIAB] OR Guyana[TIAB] OR Haiti[TIAB] OR Honduras[TIAB] OR India[TIAB] OR Indonesia[TIAB] OR Iran[TIAB] OR Iraq[TIAB] OR Jamaica[TIAB] OR Jordan[TIAB] OR Kazakhstan[TIAB] OR Kenya[TIAB] OR Kiribati[TIAB] OR Republic of Korea[TIAB] OR North Korea[TIAB] OR DPRK[TIAB] OR Kosovo[TIAB] OR Kyrgyzstan[TIAB] OR Kirghizstan[TIAB] OR Kirgizstan[TIAB] OR Kirghizia[TIAB] OR Kirgizia[TIAB] OR Kyrgyz[TIAB] OR Kirghiz[TIAB] OR Kyrgyz Republic[TIAB] OR Lao[TIAB] OR Laos[TIAB] OR Latvia[TIAB] OR Lebanon[TIAB] OR Lesotho[TIAB] OR Basutoland[TIAB] OR Liberia[TIAB] OR Libya[TIAB] OR Lithuania[TIAB] OR Macedonia[TIAB] OR Madagascar[TIAB] OR Malagasy Republic[TIAB] OR Malawi[TIAB] OR Nyasaland[TIAB] OR Malaysia[TIAB] OR Malaya[TIAB] OR Malay[TIAB] OR Maldives[TIAB] OR Mali[TIAB] OR Marshall Islands[TIAB] OR Mauritania[TIAB] OR Mauritius[TIAB] OR Mayotte[TIAB] OR Mexico[TIAB] OR Micronesia[TIAB] OR Moldova[TIAB] OR Moldovia[TIAB] OR Mongolia[TIAB] OR Montenegro[TIAB] OR Morocco[TIAB] OR Mozambique[TIAB] OR Myanmar[TIAB] OR Burma[TIAB] OR Namibia[TIAB] OR Nepal[TIAB] OR Nicaragua[TIAB] OR Niger[TIAB] OR Nigeria[TIAB] OR Pakistan[TIAB] OR Palau[TIAB] OR Palestine[TIAB] OR Panama[TIAB] OR Papua New Guinea[TIAB] OR Paraguay[TIAB] OR Peru[TIAB] OR Philippines[TIAB] OR Romania[TIAB] OR Rumania[TIAB] OR Roumania[TIAB] OR Russia[TIAB] OR Russian Federation[TIAB] OR USSR[TIAB] OR Soviet Union[TIAB] OR Union of Soviet Socialist Republics[TIAB] OR Rwanda[TIAB] OR Ruanda-Urundi[TIAB] OR Samoa[TIAB] OR Samoan Islands[TIAB] OR Sao Tome[TIAB] OR Principe[TIAB] OR Senegal[TIAB] OR Serbia[TIAB] OR Montenegro[TIAB] OR Yugoslavia[TIAB] OR Seychelles[TIAB] OR Sierra Leone[TIAB] OR Solomon Islands[TIAB] OR Somalia[TIAB] OR South Africa[TIAB] OR Sri Lanka[TIAB] OR Ceylon[TIAB] OR Saint Kitts[TIAB] OR St Kitts[TIAB] OR Saint Christopher Island[TIAB] OR Nevis[TIAB] OR Saint Lucia[TIAB] OR St Lucia[TIAB] OR Saint Vincent[TIAB] OR St Vincent[TIAB] OR Grenadines[TIAB] OR Sudan[TIAB] OR Suriname[TIAB] OR Surinam[TIAB] OR Swaziland[TIAB] OR Syria[TIAB] OR Syrian Arab Republic[TIAB] OR Tajikistan[TIAB] OR Tadzhikistan[TIAB] OR Tadjikistan[TIAB] OR Tanzania[TIAB] OR Thailand[TIAB] OR Timor-Leste[TIAB] OR East Timor[TIAB] OR Togo[TIAB] OR Togolese Republic[TIAB] OR Tonga[TIAB] OR Tunisia[TIAB] OR Turkey[TIAB] OR Turkmenistan[TIAB] OR Turkmenia[TIAB] OR Tuvalu[TIAB] OR Uganda[TIAB] OR Ukraine[TIAB] OR Uruguay[TIAB] OR Uzbekistan[TIAB] OR Vanuatu[TIAB] OR New Hebrides[TIAB] OR Venezuela[TIAB] OR Vietnam[TIAB] OR Viet Nam[TIAB] OR West Bank[TIAB] OR Gaza[TIAB] OR Yemen[TIAB] OR Zambia[TIAB] OR Zimbabwe[TIAB] OR Rhodesia[TIAB]
10	1 OR 2 OR 3 OR 4 OR 5 OR 6 OR 7 OR 8 OR 9
11	Developed countr*[MH] or developed countr*[TIAB] OR developed nation*[TIAB] OR developed world[TIAB] OR first world[TIAB] OR first-world[TIAB] OR first world countr*[TIAB] OR first-world countr*[TIAB]
12	most developed countr*[TIAB] OR most developed nation*[TIAB] OR most developed world[TIAB] OR most-developed countr*[TIAB] OR most-developed nation*[TIAB] OR more-developed countr* OR more-developed nation*[TIAB] OR more developed countr*[TIAB] OR more developed nation*[TIAB]
13	over-developed countr*[TIAB] OR over developed countr*[TIAB] OR overdeveloped countr*[TIAB] OR over -developed nation*[TIAB] OR over developed nation*[TIAB] OR overdeveloped nation*[TIAB] OR over-developed world[TIAB] OR over developed world[TIAB] OR overdeveloped world[TIAB] OR over-developed econom*[TIAB] OR over developed econom*[TIAB] OR overdeveloped econom*[TIAB] OR advanced econom*[TIAB] OR advanced econom*[TIAB]

14	first world countr*[TIAB] OR first world nation*[TIAB] OR first-world countr*[TIAB] OR first-world nation*[TIAB]
15	High-income countr*[TIAB] OR high income countr*[TIAB] OR high-income nation*[TIAB] OR high-income world[TIAB] OR high income world[TIAB] OR high econom*[TIAB] OR high income econom*[TIAB] OR high-income econom*[TIAB]
16	HIC[TIAB] OR HICs[TIAB] OR HIN[TIAB] OR HINs[TIAB] OR HIW[TIAB] OR HE[TIAB] OR HEs[TIAB] OR HIE[TIAB] OR HIEs[TIAB] OR MDC[TIAB] OR MDCs[TIAB]
17	Middle east[MH] OR North Africa[MH] OR Europe[MH] OR Central Asia[MH] OR North America[MH] OR East Asia[MH] OR West Asia[MH] OR Australasia[MH]
18	Andorra[TIAB] OR Gibraltar[TIAB] OR Oman[TIAB] OR Antigua[TIAB] OR Barbuda[TIAB] OR Greece[TIAB] OR Aruba[TIAB] OR Greenland[TIAB] OR Portugal[TIAB] OR Australia[TIAB] OR Guam[TIAB] OR Puerto Rico[TIAB] OR Austria[TIAB] OR Hong Kong[TIAB] OR Qatar[TIAB] OR The Bahamas[TIAB] OR Hungary[TIAB] OR San Marino[TIAB] OR Bahrain[TIAB] OR Iceland[TIAB] OR Saudi Arabia[TIAB] OR Barbados[TIAB] OR Ireland[TIAB] OR Seychelles[TIAB] OR Belgium[TIAB] OR Isle of Man[TIAB] OR Singapore[TIAB] OR Bermuda[TIAB] OR Israel[TIAB] OR Sint Maarten[TIAB] OR British Virgin Islands[TIAB] OR Italy[TIAB] OR Slovak Republic[TIAB] OR Brunei Darussalam[TIAB] OR Japan[TIAB] OR Slovenia[TIAB] OR Canada[TIAB] OR Korea Republic[TIAB] OR Spain[TIAB] OR Cayman Islands[TIAB] OR Kuwait[TIAB] OR Saint Kitts and Nevis[TIAB] OR Channel Islands[TIAB] OR Latvia[TIAB] OR Saint Martin[TIAB] OR Chile[TIAB] OR Liechtenstein[TIAB] OR Sweden[TIAB] OR Croatia[TIAB] OR Lithuania[TIAB] OR Switzerland[TIAB] OR Curaçao[TIAB] OR Luxembourg[TIAB] OR Taiwan[TIAB] OR Cyprus[TIAB] OR Macao[TIAB] OR Trinidad and Tobago[TIAB] OR Special Administrative Region China[TIAB] OR Czech Republic[TIAB] OR Malta[TIAB] OR Turks and Caicos Islands[TIAB] OR Denmark[TIAB] OR Monaco[TIAB] OR United Arab Emirates[TIAB] OR Estonia[TIAB] OR Nauru[TIAB] OR United Kingdom[TIAB] OR England[TIAB] OR Ireland[TIAB] OR Scotland[TIAB] OR Wales[TIAB] OR Faroe Islands[TIAB] OR Netherlands[TIAB] OR United States[TIAB] OR Finland[TIAB] OR New Caledonia[TIAB] OR Uruguay[TIAB] OR France[TIAB] OR New Zealand[TIAB] OR Virgin Islands[TIAB] OR French Polynesia[TIAB] OR Northern Mariana Islands[TIAB] OR Germany[TIAB] OR Norway[TIAB]
19	11 OR 12 OR 13 OR 14 OR 15 OR 16 OR 17 OR 18
20	Disabled person[MH] OR disabled person*[TIAB] OR person with disabilit* [TIAB] OR persons with disabilit*[TIAB] OR people with disability*[TIAB] OR handicapped person*[TIAB] OR handicapped people[TIAB]
21	Physical impair*[TIAB] or physically impair*[TIAB] OR physical deficien*[TIAB] OR physically deficien*[TIAB] OR physical disab*[TIAB] OR physically disab*[TIAB] OR physical handicap*[TIAB] OR physically handicap*[TIAB] OR physically challeng*[TIAB]
23	Cerebral palsy[MH] OR Cerebral pals*[TIAB] OR spinal dysraphism[MH] OR Spina bifida[TIAB] OR muscular dystrophies[MH] OR Muscular dystroph*[TIAB] OR Arthritis[MH] OR Arthriti*[TIAB] OR Osteogenesis imperfecta[TIAB] OR Musculoskeletal Abnormalities[MH] OR Musculoskeletal abnormalit*[TIAB] OR Musculo-skeletal abnormalit*[TIAB] OR Muscular abnormalit*[TIAB] OR Skeletal abnormalit*[TIAB] OR limb abnormalit*[TIAB] OR Chronic Brain Injury[MH]OR Amputation*[TIAB] or Amputee[TIAB] OR Clubfoot[TIAB] OR Poliomyelitis[MH] OR Polio*[TIAB] OR Paraplegia[MH] OR Paraplegi*[TIAB] OR Paralys*[TIAB] OR Paralyz*[TIAB] OR Hemiplegia[MH] OR Hemiplegi*[TIAB]

24	Hearing loss[MH] OR Hearing loss*[TIAB] OR hearing impair*[TIAB] OR hearing deficien*[TIAB] OR hearing disable*[TIAB] OR hearing disabili*[TIAB] OR hearing handicap*[TIAB] OR acoustic loss*[TIAB] OR acoustic impair*[TIAB] OR acoustic deficien*[TIAB] OR acoustic disable*[TIAB] OR acoustic disabili*[TIAB] OR acoustic handicap*[TIAB] OR Deaf*[TIAB] or hearing loss[TIAB]
25	Blindness[MH] OR vision loss*[TIAB] OR vision impair*[TIAB] OR vision deficien*[TIAB] OR vision disable*[TIAB] OR vision disabili*[TIAB] OR vision handicap*[TIAB] OR visual loss*[TIAB] OR visual impair*[TIAB] OR visually impair*[TIAB] OR visual deficien*[TIAB] OR visually deficien*[TIAB] OR visual disable*[TIAB] OR visually disable*[TIAB] OR visual disabili*[TIAB] OR visually disabili*[TIAB] OR visual handicap*[TIAB] OR visually handicap*[TIAB] OR low vision[TIAB] OR reduced vision[TIAB] OR (blind*[TIAB] NOT double blind*[TIAB] NOT blinding[TIAB] NOT triple blind*[TIAB])
26	Mental disorder*[TIAB] OR Schizophreni*[TIAB] OR Psychosis[TIAB] OR psychoses[TIAB] OR Psychotic Disorder*[TIAB] OR Schizoaffective Disorder*[TIAB] OR Schizophreniform Disorder*[TIAB] OR schizophrenia and disorders with psychotic features[MH] OR Dementia*[TIAB] OR Alzheimer*[TIAB]
27	intellectual illness*[TIAB] OR intellectual impair*[TIAB] OR intellectual deficien*[TIAB] OR intellectual disable*[TIAB] OR intellectual disabili*[TIAB] OR intellectual handicap*[TIAB] OR intellectual retard*[TIAB] OR mental ill[TIAB] OR mentally ill[TIAB]OR mental illness*[TIAB] OR mental impair*[TIAB] OR mentally impair*[TIAB] OR mental deficien*[TIAB] OR mentally deficien*[TIAB] OR mental disable*[TIAB] OR mentally disable*[TIAB] OR mental disabili*[TIAB] OR mental handicap*[TIAB] OR mentally handicap*[TIAB] OR developmental impair*[TIAB] OR developmentally impair*[TIAB] OR developmental deficien*[TIAB] OR developmentally deficien*[TIAB] OR developmental disable*[TIAB] OR developmentally disable*[TIAB] OR developmental disabili*[TIAB] OR developmentally disabili*[TIAB] OR developmental handicap*[TIAB] OR developmentally handicap*[TIAB] OR developmental retard*[TIAB] OR developmentally retard*[TIAB] OR psychological ill[TIAB] OR psychologically ill[TIAB] OR psychological illness*[TIAB] OR psychological impair*[TIAB] OR psychologically impair*[TIAB] OR psychological deficien*[TIAB] OR psychologically deficien*[TIAB] OR psychological disable*[TIAB] OR psychologically disable*[TIAB] OR psychological disabili*[TIAB] OR psychological handicap*[TIAB] OR psychologically handicap*[TIAB]
28	Learning disorders[MH] OR learning disorder*[TIAB] OR communication disorders[MH] OR communication disorder*[TIAB] OR language disorder*[TIAB] OR speech disorder*[TIAB] OR speech disorder*[TIAB]
29	Pervasive Child Development Disorders[MH] OR autistic[TIAB] OR autism[TIAB] OR asperger*[TIAB] or dyslexi*[TIAB] OR Down's Syndrome[TIAB] OR Down Syndrome[TIAB] OR Mongolism[TIAB] or Trisomy 21[TIAB]
30	21 OR 22 OR 23 OR 24 OR 25 OR 26 OR 27 OR 28 OR 29
Menstrual Hygiene Management	
31	Menstrua* OR menses OR menarche OR catamenia OR vagiosis OR candidiasis OR hygiene
32	10 OR 19 AND 30 AND 31
33	Limit 32 to English language, publication type=conference paper, journal article, non-peer reviewed publications, annual report, journal issue, thesis, miscellaneous, case reports, comparative study, evaluation studies, government publications, meta-analysis, review, systematic reviews
