# Supplementary material for: Systematic review of menstrual hygiene management requirements, its barriers and strategies for disabled people
Source: PLoS One. 2019 Feb 6;14(2):e0210974. doi: 10.1371/journal.pone.0210974 (PMC6365059; doi:10.1371/journal.pone.0210974)
Supplement: S2 Table — (PDF) [file pone.0210974.s003.pdf]

S2 Table. Quality assessment of all studies

	Altundag 2015	Carnaby 2002	Charlifue 1992	Chou 2009	Chou 2012	Patage 2015	Gold-stein 1988	Hamilton 2011	Ibralic 2010	Kirkham 2013	Kyrkou 2005	Lin 2011	Lin 2011	Mason 2007	Obaydi 2008	Perrin 1976	Rang-anath 2012	Rodgers 2005	Thapa 2017	Van der Merwe 1987	van Schroj-enstein Lantman -deValk 2011	White 2016
**Quantitative studies**																						
1. Sampling methods																						
1.1 Was the sample representative of the broader population?	3	N/A*	3	2	N/A	3	2	3	2	3	2	3	3	N/A	2	N/A	1	2	N/A	3	3	N/A
1.2 Was recruitment of participants appropriate to the study question?	3	N/A	3	3	N/A	3	3	3	2	3	3	3	3	N/A	3	N/A	2	3	N/A	3	3	N/A
1.3 Adequate sample size (>100 or sample size calculation undertaken)	2	N/A	3	2	N/A	2	2	1	2	3	2	3	3	N/A	2	N/A	1	3	N/A	1	3	N/A
1.4 Response rate reported and acceptable (≥70%)	2	N/A	3	3	N/A	1	1	1	2	2	2	3	3	N/A	2	N/A	1	2	N/A	1	3	N/A
1.5 Control group is appropriate, clearly defined (if applicable)	N/A	N/A	N/A	N/A	N/A	N/A	3	N/A	N/A	N/A	N/A	N/A	N/A	N/A	3	N/A	N/A	N/A	N/A	N/A	N/A	N/A
2. Data collection																						
2.1 Sample characteristics clearly described	1	N/A	3	3	N/A	3	3	3	2	3	1	3	3	N/A	2	N/A	1	3	N/A	3	3	N/A
2.2 Means of collecting data (e.g. assessment tool, questionnaire, etc) valid, reliable	2	N/A	3	3	N/A	3	3	3	3	3	2	3	3	N/A	3	N/A	3	2	N/A	2	2	N/A
3. Data analysis / interpretation																						
3.1 Potential confounders	1	N/A	1	2	N/A	2	2	1	2	2	2	2	2	N/A	2		1	1		1	2	

taken into account during the analysis and interpretation																						
3.2 Tests for statistical significance undertaken, presented	2	N/A	3	3	N/A	1	3	3	3	3	1	3	3	N/A	3		1	3		1	3	
**Qualitative studies (adapted from RATS)**																						
1. Study design																						
1.1 Study design is appropriate to the research question	N/A	1	N/A	N/A	3	N/A	N/A	N/A	N/A	N/A	N/A	N/A	N/A	3	N/A	2	N/A	N/A	3	N/A	N/A	3
1.1.2 Could a quantitative approach have worked better?	N/A	1	N/A	N/A	3	N/A	N/A	N/A	N/A	N/A	N/A	N/A	N/A	3	N/A	2	N/A	N/A	3	N/A	N/A	3
1.1.3 Justified why a particular method was chosen, e.g.: a)Interviews: experience, perceptions, behaviour, practice; b) Focus groups: group dynamics, convenience, non-sensitive topics; c) Ethnography: culture, organizational behaviour, interaction	N/A	1	N/A	N/A	3	N/A	N/A	N/A	N/A	N/A	N/A	N/A	N/A	3	N/A	2	N/A	N/A	3	N/A	N/A	3
2. Sampling methods																						
2.1 Criteria for selecting study sample is appropriate, e.g. purposive (diversity of opinion), random (generalizable	N/A	2	N/A	N/A	3	N/A	N/A	N/A	N/A	N/A	N/A	N/A	N/A	3	N/A	2	N/A	N/A	3	N/A	N/A	3

to broader population), volunteer (hard to reach groups)																						
2.2 Details given of how recruitment was conducted and by whom	N/A	2	N/A	N/A	3	N/A	N/A	N/A	N/A	N/A	N/A	N/A	N/A	3	N/A	3	N/A	N/A	3	N/A	N/A	3
2.3 Details given on who chose not to participate and why	N/A	2	N/A	N/A	3	N/A	N/A	N/A	N/A	N/A	N/A	N/A	N/A	2	N/A	2	N/A	N/A	3	N/A	N/A	2
3. Data collection																						
3.1 Collection of data is comprehensive and appropriate. E.g. a) Was the study setting appropriate? E.g. protection of confidentiality for sensitive discussions; b) Is the role of the researcher(s) appropriate? How might they bias the study and results? e.g. Do researchers occupy dual roles (clinician and researcher	N/A	2	N/A	N/A	3	N/A	N/A	N/A	N/A	N/A	N/A	N/A	N/A	2	N/A	2	N/A	N/A	3	N/A	N/A	3
4. Data analysis/interpretation																						
4.1 Are interpretations clearly presented and supported adequately by evidence?	N/A	3	N/A	N/A	3	N/A	N/A	N/A	N/A	N/A	N/A	N/A	N/A	2	N/A	3	N/A	N/A	3	N/A	N/A	3
4.2 Indicators of quality																						
- Description of how themes were derived from the data	N/A	2	N/A	N/A	3	N/A	N/A	N/A	N/A	N/A	N/A	N/A	N/A	3	N/A	2	N/A	N/A	3	N/A	N/A	3

(inductive or deductive)																						
- Semi quantification when appropriate	N/A	3	N/A	N/A	3	N/A	N/A	N/A	N/A	N/A	N/A	N/A	N/A	3	N/A	2	N/A	N/A	3	N/A	N/A	3
- Quote use appropriate, effective	N/A	3	N/A	N/A	3	N/A	N/A	N/A	N/A	N/A	N/A	N/A	N/A	3	N/A	3	N/A	N/A	3	N/A	N/A	3
-Analysis /presentation of negative/deviant cases, alternative explanations	N/A	2	N/A	N/A	2	N/A	N/A	N/A	N/A	N/A	N/A	N/A	N/A	2	N/A	2	N/A	N/A	2	N/A	N/A	2
-Method of reliability check (e.g. triangulation, independent review of data to contest themes)	N/A	1	N/A	N/A	3	N/A	N/A	N/A	N/A	N/A	N/A	N/A	N/A	3	N/A	1	N/A	N/A	2	N/A	N/A	2
4.3 Are findings generalizable to a broader population?	N/A	2	N/A	N/A	3	N/A	N/A	N/A	N/A	N/A	N/A	N/A	N/A	3	N/A	1	N/A	N/A	3	N/A	N/A	3
	16	27	22	21	41	18	22	18	18	22	15	23	23	38	22	29	11	19	40	15	22	39
**Grading**	+	++	++	++	++	+	++	+	+	++	+	++	++	++	++	+	-	+	++	-	++	++

*N/A marked under quantitative criteria for qualitative studies and vice versa.

RUBRIC

++ Low risk of bias: All or almost of the above criteria were fulfilled, and those that were not fulfilled were thought unlikely to alter the conclusions of the study.

+ Medium risk of bias: Some of the above criteria were fulfilled, and those not fulfilled were thought unlikely to alter the conclusions of the study.

- High risk of bias: Few or no criteria were fulfilled, and the conclusions of the study were thought likely or very likely to alter with their inclusion.
