# Supplementary material for: Systematic review of menstrual hygiene management requirements, its barriers and strategies for disabled people
Source: PLoS One. 2019 Feb 6;14(2):e0210974. doi: 10.1371/journal.pone.0210974 (PMC6365059; doi:10.1371/journal.pone.0210974)
Supplement: S3 Table — (PDF) [file pone.0210974.s004.pdf]

S3 Table. Summarised data extraction table

Citation	Title	Study location	Study design	Source of participants	Disability group	Means of assessing disability	Disabled person (n)	Carer (n)	Carer type	Theme	Overview of results
**Altundag et al (2015)**	Teaching menstrual care skills to intellectually disabled female students	Turkey (LMIC)	Quasi-experiment	Institution	Intellectual	Clinical	54	Number not specified in paper	Not specified in paper	1. MHM training and support for people with intellectual impairments	1. MHM training using a doll helped people with ID manage more independently
**Carnaby et al (2002)**	Getting personal: an exploratory study of intimate and personal care provision for people with profound and multiple intellectual disabilities	UK (HIC)	Qualitative	Residential home	Intellectual		15	Number not specified in paper	Professional	1. Concerns of carers of people with intellectual impairments<br>2. MHM training and support for carers	1. Day care staff disliked menstrual care tasks most. Residential service staff rated menstrual care just behind giving enemas (day care staff don't give enemas). Intimate care carries social taboos.<br>2. Carers noted a lack of training / standards of care for intimate and personal care tasks and requested formalised guidance.
**Charlifue et al (1992)**	Sexual issues of women with spinal cord injuries	HIC	Quantitative	Hospital	Physical	Clinical	231	Number not specified in paper	Not specified in paper	1. Menstrual product acceptability for people with physical impairments	1. 19% users reported problem using a menstrual product.<br>2. >50% needed assistance MHM<br>3. 43% reported bowel / bladder changes when menstruating
**Chou et al (2009)**	Prevalence and severity of menstrual symptoms among institutionalised women with an intellectual disability	Taiwan (HIC)	Quantitative	Welfare institution	Intellectual	Means of assessing disability not specified in paper	92	Number not specified in paper	Not specified in paper	1. Pre-menstrual symptoms and communication difficulties experienced by people with intellectual impairments	1. Level of ID is significantly associated with the domain of Water Retention (p 5 .01) and Negative Affect (p 5 .001); and women with mild/moderate ID - more likely to have a higher level of Water Retention and Negative Affect than women with severe/profound ID
**Chou et al (2012)**	Caring for a daughter with intellectual disabilities in managing menstruation: a mother's perspective	Taiwan (HIC)	Qualitative	Household	Intellectual	Means of assessing disability not specified in paper	13	12	Parent	1. Pre-menstrual symptoms and communication difficulties experienced by people with intellectual impairments<br>2. Concerns of carers of people with intellectual impairments<br>3. MHM training and support for carers<br>4. Menstrual suppression	1. Mothers who can't communicate with daughters rely on changes in behaviour to predict period / PMS.<br>2. Carers reported challenges of sourcing appropriate sanitary protection and getting daughters to use them. Mens product: expensive, not effective, daughters take them off<br>3. No mother had had access to information about how to manage menstruation. Mens care viewed as a private issue. Mothers used fate as a coping strategy instead of seeking assistance from professionals.<br>2. Most mothers were advised to sterilise their daughters by medical practitioners, health practitioners, professionals from a service agency. Reasons given for suggesting sterilisation: eliminating menstrual care ('tedious task'), hygiene, preventing pregnancy if daughter raped. Mothers viewed regular menstruation positively - sign of good health
**Goldstein (1988)**	Menarche, menstruation, sexual relations and contraception of adolescent females with Down syndrome	Denmark (HIC)	Quantitative	Households and hospital	Intellectual	Clinical	15	Number not specified in paper	Not specified in paper	1. Pre-menstrual symptoms and communication difficulties experienced by people with intellectual impairments	1. 20% of DS did not change their sanitary pads themselves. All controls did this independently.<br>2. No significant differences between test and control across: average menarche age, regularity of menstruation, duration of bleeding, length of menstrual cycle, PMS.
**Hamilton et al (2011)**	Autism spectrum disorders and menstruation	America (HIC)	Quantitative	Household	Intellectual	Self-reported	124	Number not specified in paper	Not specified in paper	1. Pre-menstrual symptoms and communication difficulties experienced by people with intellectual impairments	Differences between Asperger syndrome (AS) and autistic spectrum disorder (ASD) groups: ASD reported more hygiene issues; ASs reported more dysmenorrhea symptoms than ASD (could be ability to understand and communicate)
**Ibralic (2010)**	Age at menarche and premenstrual syndrome in adolescent girls with intellectual disability in Bosnia and Herzegovina	Bosnia and Herzegovina (LMIC)	Quantitative	Hospital	Intellectual	Clinical	31	Number not specified in paper	Not specified in paper	1. Pre-menstrual symptoms and communication difficulties experienced by people with intellectual impairments	1. No statistical difference between mean age of menarche between ID and control; no overall difference in physical symptoms between test and control.<br>2. Greater variability in the age at menarche between test and controls

Citation	Title	Study location	Study design	Source of participants	Disability group	Means of assessing disability	Disabled person (n)	Carer (n)	Carer type	Theme	Overview of results
**Kirkham et al (2013)**	Trends in menstrual concerns and suppression in adolescents with developmental disabilities	Canada (HIC)	Quantitative	Hospital	Intellectual	Clinical	300	Number not specified in paper	Professional and parent	1. Concerns of carers of people with intellectual impairments	Menstruation - one of top 3 concerns for carers; menstrual suppression was the most common concern of carers
**Kyrkou (2005)**	Health issues and quality of life in women with intellectual disability	Australia and New Zealand (HIC)	Quantitative	Household	Intellectual	Means of assessing disability not specified in paper	24	Number not specified in paper	Parent	1. Pre-menstrual symptoms and communication difficulties experienced by people with intellectual impairments	1. Period pain: 62.5% women/w autism; 75% of women/w DS, 100% AS appear to have pain, compared to 50% of the general population.<br>2. Ability to report pain: 8 women/ DS could point to or state where they have pain, even those with limited communication skills. Zero women/w autism could; 1 woman/w AS could even though all women/w AS had good communication skills.<br>3. Behaviour / symptoms of PMS: facial pallor, restlessness, excessive talking, sleep and appetite disturbance, wanting to be left alone, or exaggeration of the usual symptoms of their disability<br>4. PMS rates: General population 20-80% have mild–moderate PMS, 2-10% have PMS that interferes with usual lifestyles. Over 75 % of women/w DS complained of increased irritability. Over 75 % of women/w autism complained of irritability and lethargy.
**Lin et al (2011a)**	Predictors of caregiver supportive behaviors towards reproductive health care for women with intellectual disabilities	Taiwan (HIC)	Quantitative	Welfare institution	Intellectual	Means of assessing disability not specified in paper	0	1152	Professional	1. MHM training and support for carers	Caregiver supportive behavioural score towards reproductive health care for women with ID: menstrual issues = 8.03 3.98; sex education = 8.18 4.14; menopause issues = 7.81 4.61; preventive health services = 6.07 4.64.
**Lin et al (2011b)**	Caregiver awareness of reproductive health issues for women with intellectual disabilities	Taiwan (HIC)	Quantitative	Welfare institution	Intellectual	Means of assessing disability not specified in paper	0	1152	Professional	1. MHM training and support for carers	1. Almost 1/4 of carers unfamiliar with menstruation. Respondents responded incorrectly to statements such as “menstrual pain is one of the symptoms of reproductive diseases” (36.7%); “it is abnormal to menstruate before 16 years of age” (37.1%).
**Mason et al (2007)**	An Exploration of Issues around Menstruation for Women with Down Syndrome and their Carers	England (HIC)	Qualitative	Household	Intellectual	Means of assessing disability not specified in paper	6	53	Parent	1. Concerns of carers of people with intellectual impairments<br>2. Menstrual product acceptability for people with intellectual impairments<br>3. MHM training and support for people with intellectual impairments	1. Women/w DS who'd been given info re MHM before puberty were better able to cope. 2. People with ID: limited / confused understanding re the reproductive function of menstruation (could be because they've never been taught). Often menstruation means stomach ache and medication to the ppl with DS. Many mothers didn't know how much their daughter's knew about MHM. Lack of understanding / information leads to fear, shock and distress. Mother's surprised when daughters started periods<br>2. Ppl with IDs didn't like wearing a mens product. Some mothers reported daughter's mens being heavier, ie 'flooding', needing 'supersized pads' (but there's no control group)<br>3. Daughter's dislike for wearing a mens product. Some mothers found mens leakages distressing. Worried daughters won't follow social norms (ie showing mens blood in public)<br>4. Some mothers tracked mens with daughter on calendar - this helped some daughters to prepare for mens. Mothers who made MHM a routine (ie changing pad at specific times in the day irrespective of need) helped daughter's awareness and reduced leakages. Many people with ID needed constant reminding re MHM

Citation	Title	Study location	Study design	Source of participants	Disability group	Means of assessing disability	Disabled person (n)	Carer (n)	Carer type	Theme	Overview of results
**Obaydi et al (2008)**	Prevalence of premenstrual syndrome in autism: a prospective observer-rated study	England (HIC)	Qualitative	Households and hospital	Intellectual	Clinical	26	Number not specified in paper	Professional	1. Pre-menstrual symptoms and communication difficulties experienced by people with intellectual impairments	1. PMS rates: 92% in the autism group; 11% in the control group; difference is highly statistically significant (P<0.000 001)<br>2. Symptoms which had a marked increase autistic spectrum disorder group: affective lability; anger or irritability; clumsiness; anxiety or tension; depressed mood; impairment of work, performance, social activities or relationships; social withdrawal, isolation and decreased interest in usual activities; decreased<br>concentration; temper tantrums; physical aggression; self harm; stereotypies or repetitive movements; destructive behaviour; hypersomnia; insomnia; a change in appetite or a specific food craving; and headache.
**Patage et al (2015)**	Reproductive and sexual health needs among differently abled individuals in the rural field practice area of a medical college in Karnataka, India	India (LMIC)	Quantitative	Household	Mixed	Government lists	198	Number not specified in paper	Not specified in paper	1. Menstrual product acceptability for people with intellectual impairments	MH product used: Sanitary napkin: 54.67; Cloth: 38.67%
**Perrin et al (1976)**	A considered approach to sterilization of mentally retarded youth	America (HIC)	Qualitative	Hospital	Intellectual	Clinical	20	Number not specified in paper	Not specified in paper	1. Pre-menstrual symptoms and communication difficulties experienced by people with intellectual impairments<br>2. Menstrual suppression	1. Mens 'behaviours': increased hyperactivity, withdrawal, fear, refusing to eat<br>2. Inability to cope during mens so girls were excluded from school during mens<br>3. Carers coping strategies: keep daughters at home during mens<br>4. Lack of definitive laws on sterilsation: Human rights should be extend to the disabled person and the parents; Clear laws are needed for the protection of all involved including medical practitioners who could be sued.
**Ranganath et al (2012)**	Menstrual history in women with Down Syndrome - A review	India (LMIC)	Quantitative	Hospital (outpatients)	Intellectual	Self-reported	0	10	Parent	1. Pre-menstrual symptoms and communication difficulties experienced by people with intellectual impairments<br>2. Menstrual product acceptability for people with intellectual impairments	1. 77.5% needed help changing sanitary pads.<br>2. None reported pain, PMS
**Rodgers et al (2005)**	The nature and extent of help given to women with intellectual disabilities to manage menstruation	UK (HIC)	Quantitative	Household and hospital	Intellectual	Self-reported	452	217	Professional and parent	1. MHM training and support for people with intellectual impairments	1. 29% no one had ever tried to teach ppl with an ID. No relationship between the level of IQ and whether anyone had tried to teach them.<br>2. Menstrual care provided does not correlate to severity of ID: Mild ID – carer does the task: Keeping clean during period: 23%; Obtaining sanitary pads / tampons: 13%; Changing sanitary pads / tampons: 7%; Disposing of sanitary pads / tampons: 7%. Severe / profound IDs – carer does not do the task: Keeping clean during period: 9%; Obtaining sanitary pads / tampons: 6%; Changing sanitary pads / tampons: 16%; Disposing of sanitary pads / tampons: 14%

Citation	Title	Study location	Study design	Source of participants	Disability group	Means of assessing disability	Disabled person (n)	Carer (n)	Carer type	Theme	Overview of results
**Thapa et al (2017)**	Lost in transition: menstrual experiences of intellectually disabled school-going adolescents in Delhi, India	India (LMIC)	Qualitative	Institutions (schools)	Intellectual	Clinical	0	23	Parent	1. Concerns of carers of people with intellectual impairments<br>2. Pre-menstrual symptoms and communication difficulties experienced by people with intellectual impairments<br>3. MHM training and support for people with intellectual impairments<br>4. MHM training and support for carers<br>5. Menstrual suppression	1.Daughters refusal to wear mens product and constantly coercing daughters to wear; limited ability to follow social / cultural norms. All cause stress, frustration, anxiety. Carers coping strategies: limit their daughter's physical movements because they need to be nearby to manage their menstruation, and to protect them from walking around with soiled clothes.<br>2. Carers said difficulties communicating is a major barrier. Ppl with IDs are unable to understand menstrual cramps or explain when in pain. Result in irritability, anger<br>3. Assumption that ppl with IDs can't understand anything so they don't get training in MHM. Ppl with IDs didnt' know how to maintain hygiene during mens, or the importance of it.<br>4. No training given to mothers. Leads to feelings of being overwhelmed. Mothers are the sole carers for MH. View it as a private issue<br>5. Reasons for sterilising daughters: drudgery of MH tasks, fear for who will care for her daughter in the future, seeing no benefit for daughters, fear of unwanted pregnancies. Levels of satisfaction with sterilisation was high. Mothers (are in high income brackets) live in urban areas with access to private medical centres and can afford the operation
**Van der Merwe (1987)**	Sterilization of mentally retarded persons	South Africa (LMIC)	Quantitative	Hospital	Mixed	Means of assessing disability not specified in paper	152	Number not specified in paper	Not specified in paper	1. Menstrual suppression	After the hysterectomy carers reported: 92% felt it was good that she isn't menstruating anymore; 98% would repeat the operation
**van Schrojenstein Lantman-deValk et al (2011)**	The use of contraception by women with intellectual disabilities	Netherlands (HIC)	Quantitative	Service provider	Intellectual	Government lists	234	Number not specified in paper	Not specified in paper	1. Pre-menstrual symptoms and communication difficulties experienced by people with intellectual impairments<br>2. Menstrual suppression	48% (n = 112) of women used contraception. Reasons: problems with menstruation, behaviour and/or prevention of pregnancy. Mens related reasons for using contraception include PMS, irregular mens, heavy mens, dymenorrhoea, metrorrhagia (57% n=35). 39% n=24 women took contraception becuase of problems with behaviour. This includes 38% n=9 of those becuase of mood changes before and during mens
**White et al (2016)**	A qualitative study of barriers to accessing water, sanitation and hygiene for disabled people in Malawi	Malawi (LMIC)	Qualitative	Household	Mixed	Self-reported	36	15	Parent	1. Menstrual product acceptability for people with physical impairments<br>2. MHM training and support for people with intellectual impairments	1. Challenges for people with visual impairments: may be unable to: respond to the visual cues of menstruation making it harder to manage, identify when their period began and ended.<br>2. Challenges for wheelchair users: discomfort from always being seated; blood can stain clothes.<br>3. Challenges for ppl with IDs: often unable to manage mens independently<br>4. Mens - source of shame, worry, discomfort and embarrassment<br>5. Girls don't go to school for 1-2 weeks during mens, or drop out when they start menstruating

Abbreviations

AS	Asperger Syndrome
ASD	Autistic Spectrum Disorder
DS	Down Syndrome
HIC	High income country
ID	Intellectual disability
LMIC	Low and middle income country
PMS	Pre-menstrual symptoms
Ppl	People
Mens	Menstruation
MHM	Menstrual hygiene management
